# Early Stages of Sensory Processing, but Not Semantic Integration, Are Altered in Dyslexic Adults

**DOI:** 10.3389/fpsyg.2016.00430

**Published:** 2016-04-21

**Authors:** Patrícia B. Silva, Karen Ueki, Darlene G. Oliveira, Paulo S. Boggio, Elizeu C. Macedo

**Affiliations:** Social and Cognitive Neuroscience Laboratory and Developmental Disorders Program, Center for Health and Biological Sciences, Mackenzie Presbyterian UniversitySao Paulo, Brazil

**Keywords:** developmental dyslexia, event-related potentials, semantic processing, N400 component, P100 component

## Abstract

The aim of this study was to verify which stages of language processing are impaired in individuals with dyslexia. For this, a visual-auditory crossmodal task with semantic judgment was used. The P100 potentials were chosen, related to visual processing and initial integration, and N400 potentials related to semantic processing. Based on visual-auditory crossmodal studies, it is understood that dyslexic individuals present impairments in the integration of these two types of tasks and impairments in processing spoken and musical auditory information. The present study sought to investigate and compare the performance of 32 adult participants (14 individuals with dyslexia), in semantic processing tasks in two situations with auditory stimuli: sentences and music, with integrated visual stimuli (pictures). From the analysis of the accuracy, both the sentence and the music blocks showed significant effects on the congruency variable, with both groups having higher scores for the incongruent items than for the congruent ones. Furthermore, there was also a group effect when the priming was music, with the dyslexic group showing an inferior performance to the control group, demonstrating greater impairments in processing when the priming was music. Regarding the reaction time variable, a group effect in music and sentence priming was found, with the dyslexic group being slower than the control group. The N400 and P100 components were analyzed. In items with judgment and music priming, a group effect was observed for the amplitude of the P100, with higher means produced by individuals with dyslexia, corroborating the literature that individuals with dyslexia have difficulties in early information processing. A congruency effect was observed in the items with music priming, with greater P100 amplitudes found in incongruous situations. Analyses of the N400 component showed the congruency effect for amplitude in both types of priming, with the mean amplitude for incongruent items being greater than that of the congruent items. Electrophysiological findings were corroborated by the N400 literature and showed that the semantic processing of individuals with dyslexia was preserved. Furthermore, the findings indicate P100 visual sensory processing deficits in the dyslexic group and may suggest difficulty in the sensory stimuli process.

## Introduction

Developmental dyslexia is a learning disorder in which the essential feature is a deficit in reading skills. According to the International Classification of Diseases (ICD-10), Developmental dyslexia is classified as a Specific Reading Disorder, included in the category of specific developmental disorders of learning disability (World Health Organization, 1992).

Developmental dyslexia (DD) is the most common learning disability and affects reading accuracy and fluency. These deficits are usually related to phonological decoding impairments, which are verified by low scores of adult individuals with dyslexia during phonological awareness tasks (Lyon, 1995, 2003; Coltheart and Jackson, 1998). In general, the core language deficits of dyslexia are related to phonological processing, with these individuals presenting processing similar to normal readers in tasks of semantic and pragmatic skills.

According to Gregorie and Pierart (1997), individuals with dyslexia present slow and painful reading, often making mistakes, such as reversing letters and syllables, auditory confusion, confusion of visually similar letters, and omission and addition of letters, syllables, and sounds. This demonstrates that both the oral language and the written word are connected to the phonological structure of speech, with the hypothesis that targeting in expression units is also represented in print at a phonemic level through the alphabet. Therefore, the written language is developed through foundations of oral language (Fletcher, 2009).

Studies indicate structural and functional abnormalities in developmental dyslexia. Such changes are present in a wide cortical network and are mainly distributed in the left hemisphere and areas generally involved in phonological processing, which include Broca's area and the planum temporale, superior and middle temporal gyri, fusiform gyrus, angular gyrus, and supramarginal gyrus. Furthermore, functional brain imaging studies have demonstrated an involvement of the left posterior cerebral system (i.e., the involvement of the dorsal and ventral connections related to reading). Thus, in normal readers, neural activity related to reading spreads from the posterior to anterior regions of the left hemisphere. However, in people suffering from dyslexia, an observed increase in the activation regions in the front of one, or both, of the hemispheres is interpreted as an attempt to minimize the loss of the function of the posterior areas of the left hemisphere (Pekkola et al., 2006; Spironelli et al., 2008).

Electroencephalogram (EEG) recording allows for the measurement of brain electrical activity, which supports accurate inferences regarding the temporal and topographic processing of cognitive skills through cognitive event-related potentials (ERPs). Studies of ERPs with child and adult individuals with dyslexia have found that changes in latency and amplitude potential are related to visual processing (Kast et al., 2010; Dujardin et al., 2011), spelling (Taroyan and Nicolson, 2009; Waldie et al., 2012), lexical decision making (Horowitz-Kraus and Breznitz, 2008; Hasko et al., 2013), and cognition (Taroyan and Nicolson, 2009; Shaul, 2011). Such processes are involved in the recognition of words. Relationships between the ERP pattern and behavioral performance in reading have been indicated. In a lexical decision task, adult individuals with dyslexia presented lower amplitudes and higher latencies in P400 and P500, and these measures were inversely correlated with accuracy (Taroyan and Nicolson, 2009).

Individuals with dyslexia have difficulty in tasks involving synchronization between auditory and visual stimuli. Auditory processing presents difficulties in discriminating rapid temporal changes in tone, consecutive acoustic events, location of the sound source, and discrimination of sounds in noisy environments (Tallal et al., 1998). These difficulties interfere with the discrimination of speech sounds. In addition, individuals with dyslexia find it difficult to process visual stimuli related to the magnocellular pathway. This causes difficulty in performing a synchronized integration of visual and auditory stimuli (i.e., crossmodal integration; Breznitz, 2008). The synchronization hypothesis proposes that for information processing to be effective, the information must be processed and integrated through more than one modality. Sela (2014) analyzed the asynchronization hypothesis in adult individuals with dyslexia in a study with a crossmodal task. Significant differences between individuals with dyslexia and good readers were found in the synchronization of auditory and visual information. The visual information is processed in the visual cortex approximately 100–150 ms after presentation of the stimulus. Auditory information is processed by the auditory cortex approximately 70–100 ms after presentation. Thus, auditory processing is faster than visual processing. In normal readers, acceleration of visual processing and deceleration of auditory information are required so that both can be integrated and processed together. However, this pattern is not observed in those with dyslexia, as faulty processing occurs in the 150–250 ms window.

From the auditory discrimination impairments, studies have shown that discrimination and processing of musical information presents correlations with phonological processing, serving as a predictor of reading ability, both in good readers and in individuals with dyslexia. From this, language skills and musical skills can be seen to be correlated and, therefore, individuals with dyslexia may have musical discrimination impairments (Forgeard et al., 2008). This relationship between music and language is due to both being related to a person's awareness of their own sound productions. Thus, musical processing correlates with phonemic awareness and, therefore, shows impairments in individuals with dyslexia (Loui et al., 2011).

Considering that reading is a visually oriented language task for the perception of alphabetic codes, it has been suggested that individuals with dyslexia have deficits in low-level visual processing, such as alphabetic patterns (graphemes), as well as in non-linguistic stimuli (e.g., shapes, colors, lines). Mayseless and Breznitz (2010) compared the performance profile and evoked related potential (ERP) of adult individuals with dyslexia and good readers in a judgment task of real objects and pseudo-objects. A longer reaction time and lower P1 latency (80–130 ms post-stimulus) were observed in individuals with dyslexia compared to good readers. However, there was no difference in the accuracy of the judgments. Both groups presented higher accuracy in the pseudo-objects task in comparison to the real objects. Source localization analysis showed a greater difference in the activation of the right hemisphere for individuals with dyslexia while viewing pseudo-objects. This might be explained by a higher cognitive demand to perform the task in individuals with dyslexia. This supports their hypothesis regarding the lack of automaticity in the visual pathway of individuals with dyslexia, being different from that observed in good readers.

Another line of research is related to language processing. Particularly, since the seminal paper of Ganis et al. (1996), several studies have been conducted to investigate the electrophysiological basis of semantics. The so-called N400 is the main component in semantic ERP studies. This component has a negative polarity and emerges at approximately 250 ms and peaks at approximately 400 ms after the onset of the stimulus. Studies have shown that this component is markedly more negative following semantically incongruent stimuli when compared to congruent stimuli and has been commonly elicited using verbal tasks (for example, word-pairs) and sentences that end with a related or unrelated target word (Ganis et al., 1996; Lau et al., 2008). Koelsch et al. (2004) showed that the processing of the meaning of target words might be similar for both language and music.

According to Duncan et al. (2009), for adequate semantic processing, sensory decoding must occur properly. Therefore, it can be predicted that semantic operations will produce a smaller or more delayed N400 in individuals with dyslexia compared to good readers. However, an abnormality in the N400 effect with semantic manipulation does not provide information about the nature of a language deficit, as it may come from precedent steps or access to word meaning. Thus, the presence of the N400 effect may be related to impaired access to the meaning of words or difficulty in the early stages of phonological processing and/or spelling of verbal stimuli that are presented visually, as shown by the causal hypothesis of dyslexia.

Robichon et al. (2002) investigated the interaction between sensory and cognitive processing in groups of adult individuals with dyslexia and good readers in tasks of semantic integration, phonemic subtraction, and pseudoword reading. Sentences were presented in which the ends incorporated congruent or incongruent words. The words were presented at either a slow rate (700 ms) or a fast rate (100 ms) and were then manipulated to test the hypothesis that individuals with dyslexia have problems processing information when it is presented quickly. It was found that the N400 was larger for incongruent endings, however, there was no difference for the different rates of presentation. In addition, the N400 was larger for the dyslexic group in the incongruous situation than for the control group. Another effect observed in both groups was that the range of the N1-P2 complex, related to sensory processing, was larger when the rate of presentation for the stimuli was slow. The authors concluded that deficits in adult individuals with dyslexia are related to the integration of the meaning of the word in the context of sentences rather than purely sensory deficits.

These results therefore corroborate the findings of other studies, such as those of Rüsseler et al. (2007), concerning semantic integration tasks in the investigation of N400, in which this component was shown to have greater amplitude and latency in adult individuals with dyslexia. Three tasks were used in this study: semantic judgment, rhyme, and gender (syntactic) words. Good readers responded faster than individuals with dyslexia in all three tasks; however, both groups showed good performance in the three tasks. The peak amplitudes of N400 did not differ between the groups, despite the larger N400 latency in the group of individuals with dyslexia in rhyme, judgment, and semantic tasks.

The study of undergraduate adults tested hypotheses related to the influence of lexical predictions on N400 amplitudes when reading sentences that end in congruent or incongruent words. This is a type of semantic anticipation that facilitates early stages of visual and orthographic processing. The results showed that when an incorrect prediction occurs, an early negativity in the central parietal distribution reminiscent of the N400 is produced. The N400 was higher in the predicted condition than the unpredicted condition. Analyzing a sentence context that is independent of prediction produced a large central-parietal negativity that was more pronounced over the right hemisphere, reflecting differences in contextual facilitation or integration difficulty. The conclusion was that the effects of prediction occur more rapidly, by approximately 100 ms. In addition, a frontal, post-N400 positivity (PNP) was modulated by both conditions, which suggests a temporal primacy for prediction in facilitating lexical access (Brothers et al., 2015).

Considering that individuals with dyslexia have greater difficulty processing written words, this study controlled this effect by presenting nonverbal visual stimuli that could be congruent (or not) to auditory stimuli. Furthermore, considering that speech and the written language present similar structures and may be impaired in dyslexia, the attempt was made to verify the hypothesis that impairment of individuals with dyslexia would be in the early stages of language processing and not in the semantic integration. The present study compared the performance of adult individuals with dyslexia with control subjects in tasks that assess semantic processing in two situations of auditory stimuli (sentences and music) with integrated visual stimuli, in a visual-auditory crossmodal task. The P100 potentials were chosen, related to visual processing and initial integration, and N400 potentials related to semantic processing. Based on visual-auditory crossmodal studies, it is understood that dyslexic individuals present impairments in the integration of these two types of tasks. Thus, it was expected that the processing of information in the dyslexic group would occur differently to that found in the control group.

## Methods

### Participants

The study was approved by the Research Ethics Committee of the Mackenzie Presbyterian University (No. 0416281373060). There were 32 adult participants (11 males), all of whom had a higher education degree and were right handed, with ages range from 19 to 41 years. Fourteen individuals with dyslexia (DG) and 18 control subjects (CG) (DG = 25.07 ± 4.08 vs. CG = 25.00 ± 5.09; *p* = 0.90) were assessed in neuropsychological and achievement tasks (Toledo Piza et al., 2014). The control group was paired with the dyslexic group by education level and age varying between 4 years more or 4 years less. Both groups had similar total IQ levels (116 ± 9 vs. 121 ± 9; *p* = 0.10) and execution IQ levels (120 ± 10 vs. 121 ± 9; *p* = 0.80), however, the individuals with dyslexia exhibited lower verbal IQ (112 ± 9 vs. 119 ± 9; *p* = 0.04). The control group was selected by the same school and class of individuals with dyslexia. No participant had psychiatric or neurological comorbidities. All dyslexic participants had been diagnosed during their school years and were recruited from a university database. Achievement skills were assessed using the Phonological Awareness Test (PAT) developed for adults and the Word Reading Competence Test 2 (WRCT). The WRCT is a lexical decision task in which items are presented in audio and visual format.

Differences between the groups were found in the writing, reading and phonological awareness tasks. In the writing task there was no difference [*F*_(1, 30)_ = 1.479, *p* = 0.249, *d* = 0.101] between the task execution time of the CG (5236.63 ± 599.39) and that of the DG (6381.23 ± 734.1). However, there were differences in the number of correct responses (*F* = 20.23, *p* = 0.001, *d* = 0.609) between the CG (45.11 ± 1.91) and the DG (31.5 ± 2.34). A similar pattern was found for the reading task. There was no difference in reading time [*F*_(1, 30)_ = 2.46, *p* = 0.141, *d* = 0.159] for the groups. Differences were found in relation to correct responses during the reading [*F*_(1, 30)_ = 29.31, *p* < 0.001, *d* = 0.693], with a greater number of correct responses by the CG (73.77 ± 1.31) than by the DG (62.5 ± 1.61). For the phonological awareness task, difference was found in response time [*F*_(1, 30)_ = 6.326, *p* = 0.026, *d* = 0.327], with the DG being slower (16783.53 ± 640.9) than the CG (14702.43 ± 523.3). There was no difference in the number of correct responses in the task [*F*_(1, 30)_ = 0.067, *p* = 0.80, *d* = 0.005].

### Experimental task

**Figure 1:**
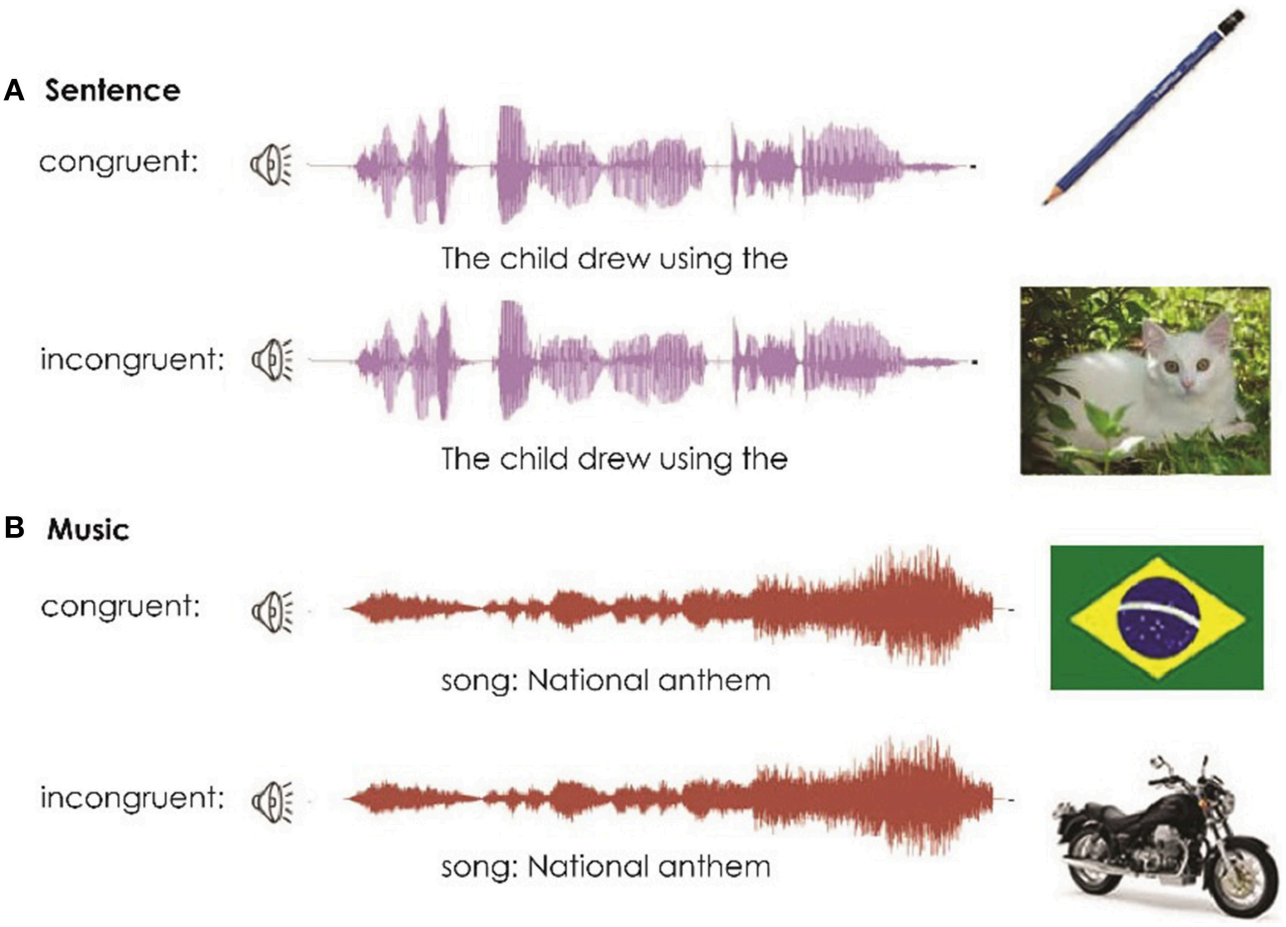
**Semantic task stimuli examples: sentence-picture pair (A, above) and music-picture pair (B, below)**.

Participants were instructed to look at a computer screen while listening to sentences (3.7 s long) or musical excerpts derived from popular television cartoons or advertisements (10 s long), which served as priming cues. Following the cue presentation, a target image was shown for 2 s and participants were instructed to evaluate whether it was related to the cue by pressing a button. Sentences were always formed by a subject followed by a verb. For example, “the child draws using a” followed by a picture of a “pencil” or “cat,” or “the girl sleeps in the” followed by a “bed” or “envelope.” Musical excerpts were chosen to be relevant to the culture. For example, the “national anthem” followed by a picture of the “national flag” or an “orange,” or “New Year's song” followed by “fireworks” or a “hairbrush.” Forty unique sentence–picture pairs and 40 unique music–picture pairs were presented in four blocks of 20 cue-target pairs, with a total of 80 balanced stimuli. Following the button-press response, a fixed cross was shown for 1 s before the beginning of the next stimulus. The order of the presentation of the stimuli was randomized and reshuffled before the beginning of each recording session. Figure 1 below shows examples of different priming cues (Figure 1).

### Event-related potential recording and analysis

The electroencephalogram was recorded continuously at 500 Hz using a 128 geodesic sensor net (EGI Inc., Eugene, Oregon, USA). Following artifact removal and re-referencing to the average, signals were epoched to −200 to 900 ms peristimulus intervals. Epochs contaminated by eye-blink, ocular movement, or other artifacts and epochs containing a difference of more than 140 mV between channels above and below the eyes, a difference of more than 55 mV between channels near the outer canthi or with one or more channels exceeding an amplitude of 200 mV were rejected. For each group (DG and CG), the different waveform for incongruent–congruent targets was extracted to obtain the P100, which was measured over occipital electrodes (right: 84, O2, 82, 88, 89, 90/left: 66, 65, 69, O1, 74, 73). On the basis of the grand average, an averaging window was defined for 50–140 ms in sentence-picture pairs and 50–160 ms in music–picture pairs. Furthermore, the N400 was extracted over central and central–parietal electrodes (right: 31, 37, 42, 54, 53, P3, 61, 60, 67, 66, 59/left: 77, 78, 79, 80, 84, 85, 86, 87, 91, P4, 93), with an averaging window of 260–460 ms in sentence-picture pairs and 300–500 ms in music–picture pairs. Repeated-measure analysis of variances was conducted, considering the P100 and N400 amplitudes as the dependent variable, the group (DG vs. CG) as the between-subjects factor and electrode (left hemisphere and right hemisphere) and congruency (congruent or incongruent) as the within-subject factor. The group vs. electrode interaction term was also modeled. Equivalent analysis of variances was also carried out on response accuracy and reaction times (RTs). Because of the different nature of the stimulus material, trials based on musical or verbal cues were analyzed separately. *Post-hoc* comparisons were performed using Fisher's LSD test. Finally, Pearson's correlation was performed between reaction time and P1 and N400 components considering congruence (congruent and incongruent) and the nature of the priming (verbal or musical).

## Results

### Verbal priming

#### Behavioral effects

Incongruent items were more accurately judged [percentage of incongruent items—98 ± 0.03 vs. congruent 87 ± 0.04; *F*_(1, 30)_ = 135.12; *p* < 0.0001; *d* = 0.818] equivalently between the two groups (*p* = 0.20). Higher RTs were observed in the DG [*F*_(1, 30)_ = 11.897; *p* = 0.002; *d* = 0.284] in congruent (DG 603.82 ± 259.91 vs. CG 405.94 ± 88.15) and incongruent items (626.59 ± 206.47 vs. 424.21 ± 98.91). There was no congruency effect [*F*_(1, 30)_ = 1.419; *p* = 0.243; *d* = 0.045] and no interaction effect [*F*_(1, 30)_ = 2.879; *p* = 0.103; *d* = 0.111].

#### P1 component

**Figure 2:**
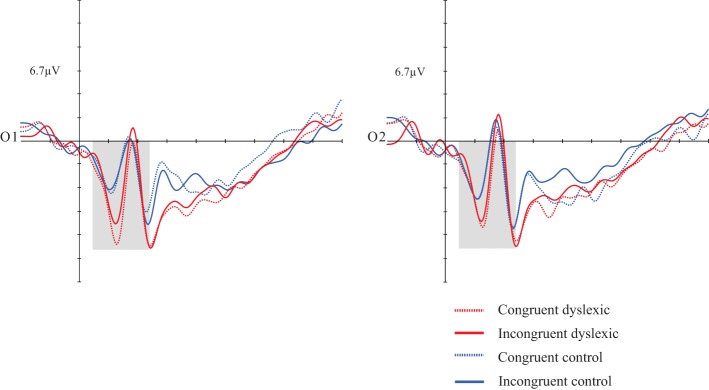
**Grand average of the P1 (50–140 ms) mean amplitude during sentence-picture pairs**. Electrodes O1 and O2 represent the occipital left and right electrodes of the EGI 128 channel system net. The DG presented higher amplitudes than the CG in congruent and incongruent trials.

Repeated measures ANOVA. Dependent variables: mean amplitude, congruency, hemisphere, and group. Interactions were also analyzed. Individuals with dyslexia exhibited greater amplitudes than those of the CG [*F*_(1, 30)_ = 4.832; *p* = 0.036; *d* = 0.139]. There was a marginal effect in the hemisphere and group interaction [*F*_(1, 30)_ = 3.892; *p* = 0.058; *d* = 0.115], with greater amplitudes in the DG in both hemispheres (see Figure 2).

#### N4 component

**Figures 3, 4:**
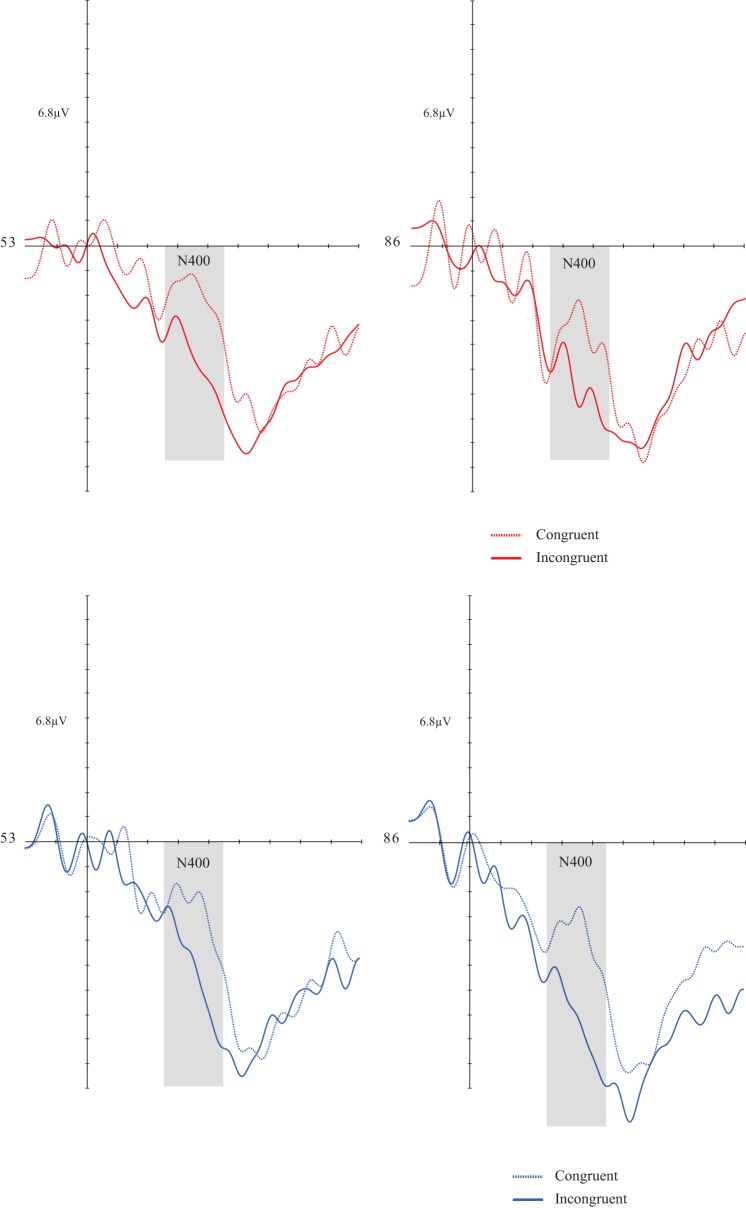
**Grand average of the N4 (260–460 ms) mean amplitude during sentence-picture pairs in the DG (left figure) and CG (right figure)**. Electrodes 53 and 86 represent the central-parietal left and right electrodes of the EGI 128 channel system net. In both groups, congruent trials evoked higher N4 amplitudes than incongruent trials.

A congruency effect [*F*_(1, 30)_ = 24.524; *p* = 0.0001; *d* = 0.450] was found. There was no significant effect for the other factors and respective interactions (Figures 3, 4).

#### Correlations between ERP and RT

Pearson's correlation analysis revealed positive significant correlations between the P1 components at the left hemisphere for both congruent and incongruent stmuli and reaction time (*r* = 0.46, *p* = 0.02 and *r* = 0.49, *p* = 0.016, respectively). No other significant correlation was observed.

### Music priming

#### Behavioral effects

As found in the verbal priming, incongruent items were more accurately judged [percentage of incongruent items 98 ± 0.02 vs. congruent 95 ± 0.06; *F*_(1, 30)_ = 9.131; *p* < 0.005; *d* = 0.233]. Individuals with dyslexia were less accurate [DG = 95 ± 0.06 vs. CG = 98 ± 0.03; *F*_(1, 30)_ = 5.917; *p* = 0.021; *d* = 0.165]. There was no interaction effect (*p* = 0.13). The DG performed poorly compared to the CG both in congruent and incongruent items [582.40 ± 188.62 vs. 380.92 ± 96.02; *F*_(1, 30)_ = 16.527; *p* = 0.0001; *d* = 0.355]. There were no congruency (*p* = 0.183) or interaction effects (*p* = 0.342).

#### P1 component

**Figures 5, 6:**
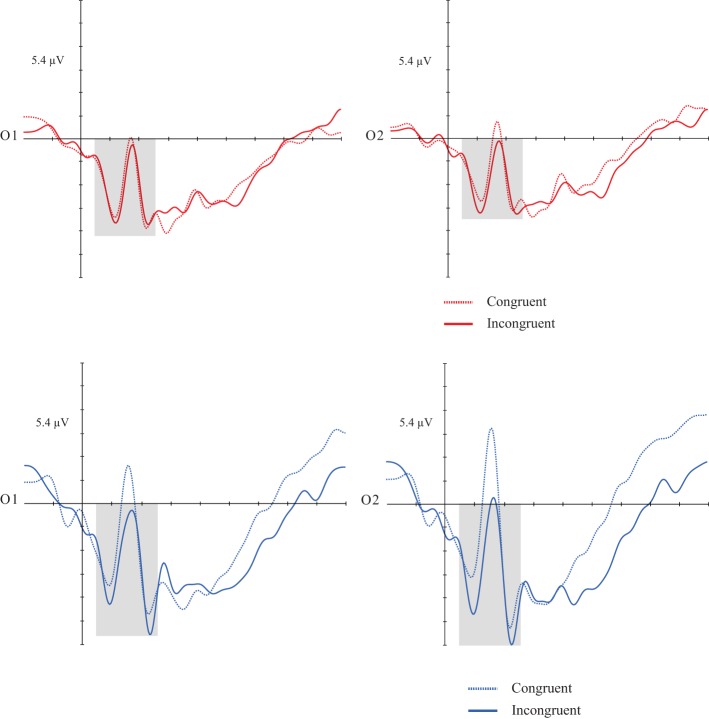
**Grand average of the P1 (50–160 ms) mean amplitude during music-picture pairs in the DG (left figure) and CG (right figure)**. Electrodes O1 and O2 represent the occipital left and right electrodes of the EGI 128 channel system net. In both groups, incongruent trials evoked higher P1 amplitudes than incongruent trials.

Repeated measures ANOVA. Dependent variables: mean amplitude, congruency, hemisphere, and group. Interactions were also analyzed. A congruency effect [*F*_(1, 30)_ = 6.600; *p* = 0.015; *d* = 0.180] was found, considering that subjects showed greater amplitudes in incongruent items than congruent ones. There was no significant effect for the others factors and respective interactions (Figures 5, 6).

#### N4 component

**Figures 7, 8:**
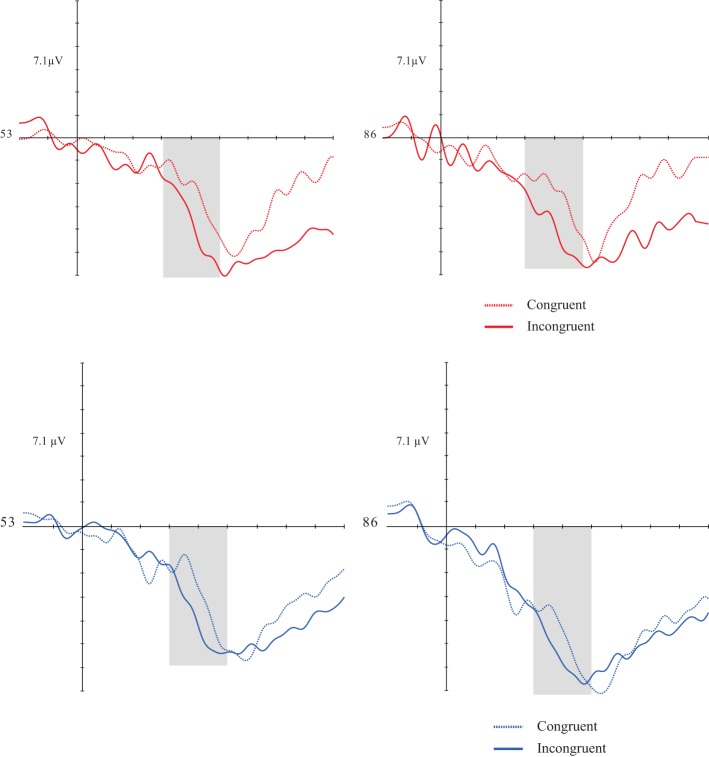
**Grand average of the P1 (300–500 ms) mean amplitude during music-picture pairs in the DG (left figure) and CG (right figure)**. Electrodes 53 and 86 represent the central-parietal left and right electrodes of the EGI 128 channel system net. In both groups, incongruent trials evoked higher N4 amplitudes than congruent trials.

As in the verbal priming, a congruency effect [*F*_(1, 30)_ = 14.931; *p* = 0.001; *d* = 0.332] was found. Both groups showed greater mean amplitudes in incongruent targets than congruent ones. There was no significant effect for the others factors and respective interactions (Figures 7, 8).

#### Correlations between ERPs and RT

For music priming, Pearson's correlation analysis did not reveal any significant correlation for both P1 and N400.

## Discussion

This study aimed to investigate the performance between adult individuals with dyslexia and good readers in two tasks related to semantic processing and to analyze the visual-auditory integration performance of the groups in the crossmodal task. Different studies usually utilize verbal stimuli through written words (Robichon et al., 2002; Sabisch et al., 2006; Rüsseler et al., 2007; Duncan et al., 2009; Schulz et al., 2009); however, this study used non-verbal visual stimuli that could be congruent to auditory stimuli. Even with the stimulus not being a word, it was expected that the groups would differ from each other, since the group of dyslexic individuals presented impairments in the visual-auditory crossmodal tasks and in both processes separately. A priming sentence and music were used considering the auditory information processing difficulties found in individuals with dyslexia. In addition, the priming music was selected due to its strong correlation with language, especially with phonological processing.

The results relative to the priming sentence and music were analyzed separately because of the difference in the presentation time of the stimuli. However, it can be seen that the behavioral data are similar in both primings. Thus, the DG presented slower auditory information processing time, regarding both the sentence and music, and slower visual-auditory crossmodal integration.

Analysis performed on the behavioral data of accuracy revealed that both items with sentence and music priming did not exhibit a significant effect for the congruency factor, as the DG and CG presented higher scores in the incongruent items than in the congruent ones. This finding corroborates the results of previous studies that used semantic judgment tasks, in which adults (Ganis et al., 1996; Robichon et al., 2002; Rüsseler et al., 2007) more easily identified items that were not related to a specific context. Furthermore, there was no difference between groups for the index of accuracy in items in which the priming was music, as the DG presented lower performance than the CG. Nevertheless, the high number of correct answers in both blocks shows that both music and language can determine the physiological indices of semantic processing (Koelsch et al., 2004).

The DG required more reaction time for a decision when the priming was verbal. As described by Pekkola et al. (2006), individuals with dyslexia rely more upon visual strategies for audiovisual processing. Thus, in this study, the visual information was presented after the auditory information, and therefore, the use of visual strategies would take longer to integrate the information.

According to Sela (2014), under the crossmodal integration condition, the presence of the auditory modality in the pre-response time frame (between 170 and 240 ms after stimulus presentation) did not increase the processing speed in the visual modality in the DG. This study found similar results, as the DG presented higher amplitudes in P100 with verbal priming. Thus, sensory information is processed differently and requires more time and cognitive effort for the integration of information. This is also corroborated by the significant positive correlation for both congruent and incongruent stimuli between P1 components at left and reaction time.

Regarding the reaction time variable, a group effect was found in music and sentence priming, in which the individuals of the DG were slower than those of the CG. This indicates that individuals with dyslexia process auditory information at a lower speed.

In the present study, significant differences were found between the groups with respect to the music priming. Individuals with dyslexia are unable to discriminate between rapid temporal changes in tones during consecutive acoustic events (Tallal et al., 1998) due to difficulties locating and integrating the origin of the sounds (Wallace and Stevenson, 2014). The results of this study corroborate other studies, considering that the auditory processing of musical items requires an analysis of tonal changes. The greater number of errors of the DG can be understood as a difficulty recognizing music integrated with images (i.e., if the acoustic information was not properly processed, a correct answer cannot be given).

The electrophysiological results correspond to different stages of cognitive processing, in which the P100 potential refers to the early sensory identification of visual stimuli, after the presentation of the auditory priming. Thus, items with a group priming effect for sentence and music were observed for the amplitude of the P100, with higher means presented by the individuals with dyslexia. Furthermore, the items with music priming presented a tendency for a hemisphere × group interaction, with greater amplitudes in the left hemisphere of the DG. A congruency effect was also observed for items with music priming, with larger P100 amplitudes in incongruous situations.

In object and pseudo-object recognition tasks, Mayseless and Breznitz (2010) found a higher P100 latency in adult individuals with dyslexia than was found in this study. However, it is possible to consider that a difficulty in decoding visual stimuli persists into adulthood, despite the adoption of compensatory strategies developed by individuals with dyslexia throughout life, as observed in the behavioral data.

Analyses of the N400 component showed a congruency effect for amplitude in both types of priming (sentence and music), with a higher mean amplitude in the incongruent items than the congruent items. This pattern corroborates the results of previous studies with adult individuals with dyslexia and control subjects in semantic processing tasks with verbal stimuli (Robichon et al., 2002; Rüsseler et al., 2007). Therefore, it can be considered that regardless of the stimuli modality (verbal or nonverbal), both groups show the same pattern of N400 amplitude in incongruent situations.

The study of Brothers et al. (2015) investigated the modulation of N400 when there are lexical predictions that facilitate the early stages of visual and orthographic processing. In adulthood, the adoption of compensatory strategies developed by individuals with dyslexia throughout life, as well as the prediction of the stimulus, can help in the discrimination of congruent stimuli. This would also help the DG at the visual processing stage and indicates that there is no difference between the groups due to the forecasting of target stimuli. Both groups, therefore, had the same pattern of N400, and there was no significant effect of the congruency factor on the accuracy of the behavioral data.

## Conclusion

The analysis of the data found in this study is exploratory, as our experimental procedure was not found in a literature survey of experiments that had the same semantic integration paradigm that we used, including a crossmodal visual-auditory task. It is therefore suggested that further studies investigate semantic processing in adult individuals with dyslexia through musical priming and the oral presentation of sentence tasks with visual target stimuli (figure). We conclude that adult individuals with dyslexia exhibit a standard N400 similar to good readers, with greater P100 amplitude.

In addition, the findings corroborate audiovisual crossmodal studies in which the synchronization of visual and auditory information of individuals with dyslexia is affected. These difficulties were presented when the priming was the sentence as well as when the priming was the music, corroborating the literature describing processing difficulties for auditory as well as musical information. In the present study, we used a cross-modal paradigm of semantic integration and synchronization. Individuals with dyslexia presented losses in the early stages of sensory processing, however, had preserved semantic integration.

## Author contributions

PD, KU—Conception, Design of the work; Acquisition of Data, Analysis of Data, Interpretation of data; Drafting the work. DO—Acquisition of Data, Analysis of Data, Interpretation of data; Drafting the work. PB—Conception, Design of the work; Analysis of Data, Interpretation of data; Drafting the work; Revising it critically for important intellectual content. EM—Conception, Design of the work; Acquisition of Data, Analysis of Data, Interpretation of data; Drafting the work; Revising it critically for important intellectual content.

## Funding

PD (Grant number: 2013/21826-4) and DO (Grant number: 2010/17123-0) were supported by Fundação de Amparo à Pesquisa do Estado de Sao Paulo (FAPESP). KU (Grant number: 09038/2010-7), PB (Grant number: 311641/2015-6), and EM (Grant number: 311479/2015-4) were supported by Conselho Nacional de Desenvolvimento Científico e Tecnológico (CNPq).

### Conflict of interest statement

The authors declare that the research was conducted in the absence of any commercial or financial relationships that could be construed as a potential conflict of interest.
